# Longitudinal Analysis (1994–2020) of Prevalence and Trends of Underweight in Polish Children

**DOI:** 10.3390/children8080643

**Published:** 2021-07-27

**Authors:** Aleksandra Lemanowicz-Kustra, Anna Borkowska, Michał Brzeziński, Adam Wyszomirski, Agnieszka Szlagatys-Sidorkiewicz

**Affiliations:** 1 Department of Pediatrics, Gastroenterology, Allergology and Nutrition, Faculty of Medicine, Medical University of Gdańsk, 80-803 Gdańsk, Poland; lemanka@gumed.edu.pl (A.L.-K.); brzezinski@gumed.edu.pl (M.B.); agnieszka.szlagatys-sidorkiewicz@gumed.edu.pl (A.S.-S.); 2 Department of Adult Neurology, Faculty of Medicine, Medical University of Gdańsk, 80-211 Gdańsk, Poland; adam.wyszomirski@gumed.edu.pl

**Keywords:** underweight, BMI, children, Poland

## Abstract

Nutritional status disorders are a worldwide problem. Approximately 5.9 million children under the age of five die each year, and 45% of these deaths are related to malnutrition. The aim of the study was to analyse the prevalence of underweight children aged between 6 and 7 years old, living in the Gdańsk, Poland, in the years 1994–2020. The anthropometric parameters of 67,842 children were analysed. BMI (Body Mass Index) value <5 percentile (pc) was defined as underweight. The BMI value was compared to the WHO (World Health Organization) centile charts and the OLAF (research project PL0080) national reference charts. The prevalence of underweight children in relation to the WHO charts was 1.9%; underweight status was found to be more significant in the group of boys (2.1%) than the group of girls (1.7%) (*p* < 0.001). According to the OLAF centile charts, the underweight figure among all of the study population was 2.1% and no statistical significance between boys (2.1%) and girls (2.0%) was found (*p* = 0.670). The occurrence of underweight indviduals in the studied group slightly increased in the years 1994–2020. We found a statistically significant increasing linear trend in the analysis of underweight children in our group (*p* < 0.001), in group of boys (*p* < 0.001), but not girls (WHO *p* = 0.603; OLAF *p* = 0.787). This points to the need to conduct regular screening systems for children and adolescents.

## 1. Introduction

Nutritional status disorders are a worldwide problem, encompassing deficiency, excess and an imbalance of energy and/or nutrient intake. Malnutrition encompasses three groups of conditions: undernutrition, which includes cachexia (low weight-for-height), stunting (low height-for-age) and underweight (low weight-for-age); excessive micronutrient intake or micronutrient deficiency; overweight, obesity and diet-related non-communicable diseases [1].

Worldwide, around 45% of deaths among children under 5 years of age are linked to undernutrition [1].

Malnutrition (often understood as underweight) can arise from many causes, including starvation, inadequate diet, severe acute (infectious) or chronic disease. Frequently, these factors can occur simultaneously. Therefore, malnutrition is a social, economic, medical and political problem [2].

Malnutrition among people in developing countries is mainly the result of poverty and insufficient food intake. The situation is worst in both South East Asia and Africa [3]. In developed countries, due to wide access to processed food and a sedentary lifestyle [4], there is a risk of deficiencies in certain vitamins and minerals and also of becoming overweight or even obese [5].

Secondary malnutrition leads to a number of changes in the human body [2,6]. These include impaired cytokine production, decreased lymphocyte count, body mass deficiency, loss of muscle mass, hypoglycaemia, impaired heart muscle function, impaired liver function, hepatic steatosis, malabsorption syndrome, abnormal intestinal flora, impaired kidney function and acute or remote CNS dysfunction (learning problems, irritability, behavioural disorders [2]. The long-term effects of malnutrition include reproductive disorders, osteoporosis and increased risk of premature death [7].

A Global Nutrition Report (2020) revealed that, in 2016, 10.3% of boys and 11% of girls between the ages of 5 and 19 were underweight across Europe [8]. The problem of malnutrition is also faced by developed countries such as the United States, where, according to the National Health and Nutrition Examination Survey (NHANES 2017–2018), 4.1% of children and adolescents aged 2–19 years are underweight [4]. Whereas in Spain, according to studies carried out in 2010, among others, 8.1% of children aged 8–11 years were found to be underweight [9].

To date, there is no gold standard method for diagnosing malnutrition, but basic anthropometric measurements, due to their availability, simplicity and low-cost, are widely used and accepted [2].

Body Mass Index (BMI), expressed as the ratio of the body mass in kilograms to the square of the height in metres, is commonly used to classify nutritional levels in children and adolescents [10]. Body mass deficiency is one of the main indicators of undernutrition in children, which is defined as a BMI below the fifth centile relevant for gender and age [10].

The prevalence of underweight children in Poland is not subject to regular nationwide screening. Numerous centres assess the nutritional status of children and adolescents; however, these epidemiological studies include children of different ages, and use different criteria for assessing body mass deficiency. Additionally, they are mostly cross-sectional studies without a long-term, continuous character [11,12]. Sometimes the studies are only conducted by survey data, and comparing their results is therefore difficult.

The aim of the study was to analyse the prevalence of body mass deficiency among children aged 6–7 years living in the municipality of Gdańsk in the period between 1994 and 2020, using two different charts: WHO and OLAF.

## 2. Materials and Methods

Within the framework of the “Healthy Life of Your Child” (“Zdrowe Życie Twojego Dziecka”) project, carried out by the Gdansk Centre for Health Promotion (Gdańskie Centrum Promocji Zdrowia), anthropometric measurements of children aged between 6 and 7 years old were taken in the years 1994–2020 (excluding 1999 and 2003—due to reorganization of the centre’s structure and lack of screening at the centre in both of these years). The programme is financed by the City of Gdańsk, and its main goal is to promote a healthy lifestyle, assess health and psychophysical development, and to identify any risks to a child’s health. It is worth mentioning that Gdańsk is a city with approximately 500,000 inhabitants, characterized by a high socioeconomic status and ethnic homogeneity.

Body height was measured with an accuracy of 1 mm (children standing barefoot with head aligned with the Frankfurt plane) and body weight was measured with an accuracy of 100 g (children were barefoot, dressed only in underwear or gym clothes). Based on the collected measurements, BMI was calculated according to the formula BMI = body mass (kg) /height (m^2^).

BMI values were related to centile charts developed by the WHO [13] and the research charts of the national research project, OLAF [14]. The study used two different sets of charts to enable the comparison of the results obtained with other studies and to analyse the data according to national standards. According to the accepted criteria, a BMI value < 5 pc was defined as underweight [10].

This study was approved by the Independent Bioethics Committee for Scientific Research at the Medical University of Gdansk (decision no. NKBBN/228/2012 and NKBBN/228−197/2014). The number from the register clinicaltrial.gov is NCT04143074.

### Statistics

Quantitative data was summarized as means and standard deviations (SD). Categorical variables were presented as percentages. Differences between the group of boys and the group of girls were evaluated using an unpaired t-test for continuous data, whereas a chi-square test was used for categorical data. The 2-tailed tests were carried out at a significance level of *p* being less than or equal to 0.05. All statistical analyses and figures were performed using the R statistical package (version 3.6.3.). The statistical significance of trends was evaluated by treating the year variable as a continuous predictor in a regression model.

## 3. Results

In this period, a total of 67,842 children were examined, including 32,572 (48.28%) girls and 35,090 (51.72%) boys. The mean age of the examined children was 6.551 ± 0.357 (Table 1). These children were born between 1987 and 2013 and approximately represent 60% of the children born in Gdańsk at that time.

The mean age, body weight, height, and BMI z-score for the WHO and OLAF are presented in Table 1. In the study group, boys had higher mean body mass (23.719 (4.344) vs. 23.099 (4.205)) than girls, as well as height (121.844 (5.534) vs. 120.865 (5.524)), and mean BMI z-score, according to both WHO criteria (−0.192 (1.244) vs. −0.096 (1.080)) and OLAF criteria (−0.096 (1.080) vs. −0.059 (0.973)).

**Figure 1:**
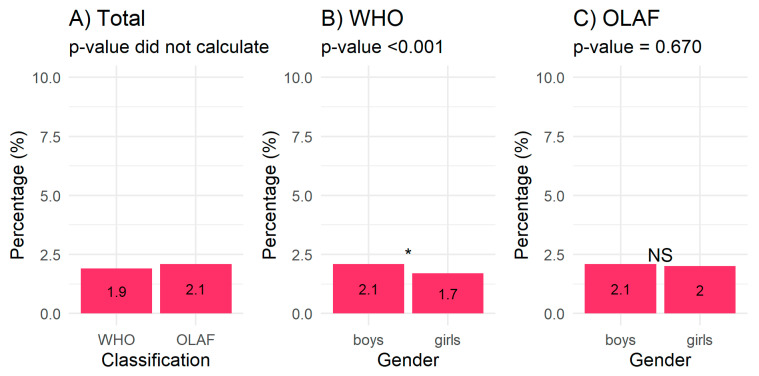
(**A**–**C**). Prevalence of underweight children by classification method and by gender according to each classification method. *—significant, NS—not significant, OLAF national research project reference charts, WHO World Health Organization.

According to the accepted criteria in relation to the WHO charts (Figure 1A), the prevalence of underweight children in the study population was 1.9%, with boys being more underweight (2.1%) than girls (1.7%); this difference was statistically significant (*p* < 0.001) (Figure 1B). On the other hand, according to the OLAF centile charts, among all examined children, 2.1% suffered from body mass deficiency (Figure 1A) and the differences between boys and girls were not statistically significant (boys 2.1%, girls 2.0%, *p* = 0.670) (Figure 1C).

**Figure 2:**
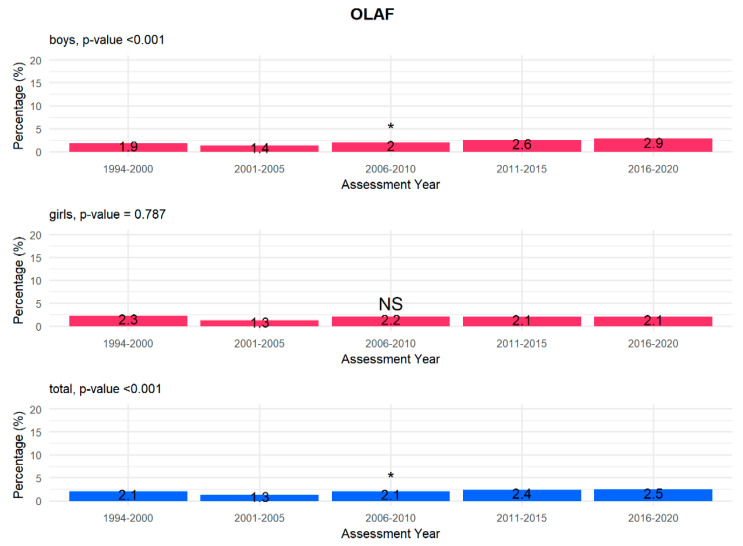
Prevalence of underweight children according to the WHO criteria by gender and year (total *p* value < 0.001, boys *p* value < 0.001, girls *p* value 0.603). *—significant linear trend, NS—not significant.

**Figure 3:**
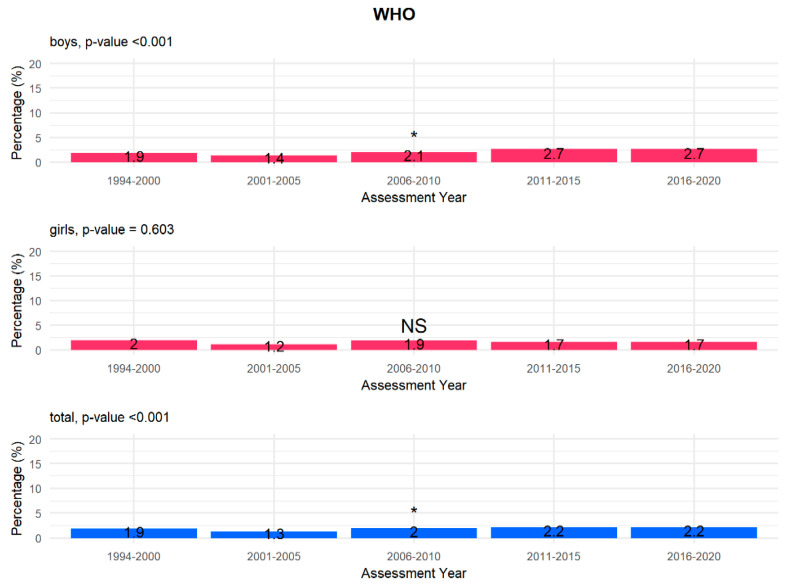
Prevalence of underweight children according to the OLAF criteria by gender and year (total *p* value < 0.001, boys *p* value < 0.001, girls *p* value 0.787). *—significant linear trend, NS—not significant.

According to gender and year of examination, our analysis shows that there is a statistically significant increasing linear trend in underweight children, defined in relation to both the WHO (Figure 2) and OLAF (Figure 3) centile charts criteria in the entire study population (*p* < 0.001) and in boys (*p* < 0.001). This trend is not observed in the girls’ group (WHO *p* = 0.603; OLAF *p* = 0.787). Data were analysed at 5-year intervals to make the results of the long-term follow-up of a large study group transparent and intelligible.

The z-score values, corresponding to the WHO and the OLAF charts, were determined for the study group of children.

In the study group, the mean z-score values were lower than 0 among boys and girls (Table 1). This may indicate that the examined population is thinner than the standard population of children aged between 6 and 7 in Poland.

When comparing mean z-score values among underweight children of different years, between genders and according to the WHO and OLAF centile charts criteria, no statistically significant differences were observed (Table 2 and Table 3).

## 4. Discussion

Nutritional status disorders are currently one of the most significant health, social and psychological problems among children. Researchers in developed countries are paying more attention to the issues that come with being overweight and obese, but much less to malnutrition and low body mass in populations under 18 years of age [15,16]. This is probably due to the epidemic of obesity among children, adolescents and adults in the USA and many Western European countries, which is associated with an increased risk of chronic diseases such as diabetes [15,16]. As a result, the problem of low body mass has been overlooked, despite the fact that it also has serious health implications [17]. Publications on obesity are based on studies of large groups in healthy populations, while publications dealing with malnutrition rarely describe particular risk groups, e.g., chronically ill children, hospitalised patients or patients with particular chronic diseases [18,19,20]. It should be emphasised that the data presented in our study are unique, due to the long-term nature of the research and the large group covered by the study.

On the basis of data collected from a representative group of children from Gdańsk (67,842), examined at the age of 6–7 years, it was found that the prevalence of body mass deficiency (BMI < 5 pc) differed depending on the centile charts used (WHO 1.9% vs. OLAF 2.1%); it also differed in relation to gender, according to the WHO (*p* < 0.001). According to the study conducted by Chabros et al. [11], the prevalence of body mass deficiency among Polish adolescents aged 11–15 years, defined as BMI < 5 pc and using centile charts developed by the Institute of Mother and Child (Polish abbrev. IMiDZ), was significantly higher in girls than it was in boys [11]. The highest percentage of underweight adolescents was found in boys aged 14 years (8.7%) and girls aged 13 years (19.3%) [11]. These results differ from those presented in this publication. This is probably due to the difference in the age of the examined persons; in the study published by Chabros, the data of adolescents were analysed. In addition, the significant difference between boys and girls obtained by the study published by Chabros deepened in subsequent years, which may be attributed to the current social trend that favours slim and very slim people, leading to more individuals with a below-average BMI.

IMiDZ centile charts were also used by Roszko-Kirpsza and co-authors, concluding that in Podlaskie Voivodeship, 24.2% of the examined children aged 1–14 years were underweight (22.2% of boys and 26.1% of girls), the cut-off point being a BMI below 10 pc [21]. Thus, almost a quarter of the children were underweight. However, the authors used a different cut-off point for their analysis, which explains the differences in the results obtained.

On the other hand, a study conducted by Kolarzyk et al. on the body mass of preschoolers found that 10.4% of children were underweight [22].

In 2012, Olejnik et al. published a study of a group of children from Podlaskie Voivodeship, which found that the percentage of malnourished children was 24.2%; in the group of children aged 1–3 years, the percentage reached an even higher figure of 32.2%. However, number of children in the study group was only 572, before splitting them into age subgroups, and therefore it may not have been representative [23].

The large differences in the number of underweight children in Poland demonstrated by various researchers are probably due to the adoption of different cut-off criteria and the use of different centile charts, sometimes adjusted only to the population in one region of the country. Moreover, the above-mentioned studies are not representative of the whole child population in Poland as only small study groups were used.

A prospective study conducted in Krakow between 1983 and 2010, covering 5245 children aged 3–18 years, also showed that method is crucial in determining body mass deficiency. Using the International Obesity Task Force (IOTF) criteria, the prevalence of body mass deficiency decreased from 10.5 to 10.3% in girls and from 8.9 to 7.5% in boys but was still higher in girls than in boys. According to the Centers of Disease Control and Prevention (CDC) criteria, the prevalence of body mass deficiency decreased from 5.1 to 4.4% and from 5.9 to 4.6%, respectively, and was slightly higher in girls [12]. It has not been clearly shown whether the problem of body mass deficiency affects girls or boys more, which is also evident in the present study, where the prevalence is higher in boys according to the WHO charts.

In our study, body mass deficiency was more prevalent when assessed using the OLAF criteria than it was when we used the WHO criteria (Figure 1A). Similarly, researchers in Iran found that differences in the prevalence of body mass deficiency depends on the selected assessment method. In the study, 70,339 children aged 0–5 years were examined. The results show that, according to the CDC centiles (<5 pc), 13.9% (8.1% boys and 5.7% girls) of the study’s participants were underweight, but only 5% (2.6% boys and 2.4% girls) were found to be underweight according to local norms and cut-off points [24].

Results similar to those described in this paper (1.9% prevalence) were obtained by authors from Kosovo, who applied a similar criteria for the diagnosis of undernutrition, although the study group was much smaller and included children aged 12–83 months [25]. Furthermore, Spanish authors proved that undernutrition is not currently a problem in the local population, occurring in less than 1% of the study population, and only in girls aged 11 years in 2.3% of the study population [26]. Differences in the prevalence of malnutrition among children depending on the criterion adopted were also observed by researchers from Ukraine, where body mass deficiency was as high as 15.2% according to the IOTF criteria, but only 4.9% for the WHO criteria 4.9% and 8.6% for the CDC criteria [27].

A meta-analysis involving 49 studies on a total of 323,420 children aged 2–18 years from 26 European countries was published in 2021. It showed that between 2000 and 2017, according to the IOTF criteria, the prevalence of body mass deficiency showed increasing trends in Eastern, Northern and Southern Europe, where the prevalence of an underweight population ranged from 9.1 to 12.0%, from 4.1 to 6.8% and from 5.8 to 6.7%, respectively. In Western Europe, the prevalence of body mass deficiency showed a decreasing trend from 14.0 to 11.8%. No significant differences were found by gender or age range [5].

In our study, on the other hand, when analysing the prevalence of underweight children according to gender and year of study, it can be observed that there is a statistically significant increasing linear trend for both the WHO (Figure 2) and the OLAF (Figure 3) centile charts criteria in the entire study population (*p* < 0.001) and in the group of boys (*p* < 0.001).

In Poland, 38 studies on the nutritional status of children and adolescents were published between 2005 and 2015 [28]. Unfortunately, despite a relatively large number of reports, they are practically incomparable. Currently, most analyses worldwide are based on the criteria recommended by the WHO, the CDC and the IOTF. To compare the nutritional status of children in Poland with global data, these assessment criteria should be used. The comparison of national data, on the other hand, should be based on current, population-representative centile charts, i.e., those developed under the OLAF project. Local scale differs from international scale because the WHO’s scale is based on measurements of children from 6 countries, while the OLAF scale is only based on the measurements of children from Poland, a developed Central European country. In addition, the height centiles of the current Polish percentile charts have a higher value compared to the WHO growth standard [14].

Our study shows that the prevalence of body mass deficiency slightly increased between 1994 and 2020. These results can be a valuable source of information for Polish authorities, paediatricians and parents. This will help guide prevention and intervention efforts. Compared to other publications, our study appears to be unique due to its long-term duration and regular observation period of a large population of children from one city.

## Figures and Tables

**Table 1:** Characteristics of the study group.

	Total	Girls	Boys	*p* Value
*n*	67,842 (100%)	32,752(48.28%)	35,090 (51.72%)	
Mean age (SD)	6.551 (0.357)	6.549 (0.356)	6.553 (0.359)	0.164
Mean weight (SD)	23.420 (4.289)	23.099 (4.205)	23.719 (4.344)	<0.001
Mean hight (SD)	121.371 (5.551)	120.865 (5.524)	121.844 (5.534)	<0.001
Mean BMI * z-score WHO ** (SD)	−0.145 (1.169)	−0.096 (1.080)	−0.192 (1.244)	<0.001
Mean BMI * z-score OLAF *** (SD)	−0.078 (0.976)	−0.059 (0.973)	−0.096 (1.080)	<0.001

Mean age, weight, height, BMI * z-score by WHO ** and OLAF ***. * BMI Body Mass Index, ** WHO World Health Organization, *** OLAF national research project reference charts.

**Table 2:** Mean z-score in underweight girls by year of study and methods.

	WHO		OLAF	
GIRLS	*n*	Mean BMI WHO z-Score (SD)	*n*	Mean BMI OLAF z-Score (SD)
1994–2000	175	−2.644 (0.974)	200	−2.824 (1.562)
2001–2005	70	−2.458 (0.492)	77	−2.541 (0.607)
2006–2010	137	−2.362 (0.379)	155	−2.426 (0.477)
2011–2015	121	−2.395 (0.379)	151	−2.413 (0.432)
2016–2020	70	−2.361 (0.317)	85	−2.395 (0.362)

**Table 3:** Mean z-score in underweight boys by year of study and methods.

	WHO		OLAF	
BOYS	*n*	Mean WHO z-Score (SD)	*n*	Mean OLAF z-Score (SD)
1994–2000	179	−2.673 (1.114)	180	−2.960 (2.866)
2001–2005	84	−2.577 (0.711)	82	−2.670 (1.082)
2006–2010	162	−2.420 (0.403)	150	−2.450 (0.422)
2011–2015	202	−2.502 (0.568)	195	−2.552 (1.131)
2016–2020	119	−2.394 (0.330)	125	−2.373 (0.346)
